# Diabetes mellitus risk in post-myocardial infarction patients: FINDRISC versus self-assessment—a cross sectional study

**DOI:** 10.1186/s12933-024-02551-1

**Published:** 2025-01-18

**Authors:** Karianne Nölken, Jakob Linseisen, Philip Raake, Christine Meisinger, Timo Schmitz

**Affiliations:** 1 Epidemiology, Medical Faculty, University of Augsburg, Augsburg, Germany; 2 Institute for Medical Information Processing, Biometry and Epidemiology (IBE), Faculty of Medicine, LMU, Munich, Germany; 3 Pettenkofer School of Public Health, Munich, Germany; 4 Department of Cardiology, Respiratory Medicine and Intensive Care, University Hospital Augsburg, Augsburg, Germany

**Keywords:** Myocardial infarction, Diabetes Mellitus, FINDRISC, Self-perception, Risk score

## Abstract

**Background:**

The aim of this study was to investigate the difference between perceived and calculated diabetes risks among post-myocardial infarction (AMI) patients using the Finnish Diabetes Risk Score (FINDRISC).

**Methods:**

The study population includes individuals from the Myocardial Infarction Registry in Augsburg, Germany, who had not been previously diagnosed with diabetes and who received a postal follow-up questionnaire after hospital discharge. A total of 466 participants completed the questionnaire, which collected information on age, sex, body mass index (BMI), waist circumference, physical activity, eating habits, use of antihypertensive medication, previous hyperglycemia, and family history of diabetes. These factors are components of the FINDRISC score, which estimates the likelihood of developing diabetes within the next 10 years. Furthermore, the participants were asked, how they would rate their personal risk to develop diabetes. The analysis focused on determining how many post-AMI patients correctly estimated their diabetes risk compared to the risk calculated by the FINDRISC score. Furthermore, multivariable logistic regression was used to analyze determinants associated with risk underestimation.

**Results:**

Results showed that a significant proportion of the AMI population (58%) underestimated their diabetes risk. This underestimation was significantly associated with older age, higher BMI, greater waist circumference, elevated blood glucose levels, use of antihypertensive medication and a family history of diabetes. Higher education contributed to more accurate risk perception.

**Conclusion:**

This study contributes to the understanding of diabetes risk perception in AMI patients and highlights the need for improving diabetes risk awareness through targeted education and healthcare communication interventions. These efforts can help patients understand their health risks, which improves health outcomes and preventive care.

**Supplementary Information:**

The online version contains supplementary material available at 10.1186/s12933-024-02551-1.

## Introduction

Cardiovascular disease (CVD) and diabetes mellitus are two of the most common public health problems worldwide, affecting millions of people each year [1, 2]. Among patients with acute myocardial infarction (AMI), multimorbidity is prevalent, with type 2 diabetes mellitus being one of the most common comorbidities and risk factor for poor prognosis across all age groups. This coexistence notably worsens prognosis and complicates disease management [3, 4].

Therefore, the Finnish Diabetes Risk Score (FINDRISC) was developed as screening tool for drug-treated diabetes and prediction of future diabetes risk without the need of laboratory tests. The method is based on readily available parameters: age, BMI, waist circumference, regular physical activity, fruit and vegetable intake, use of antihypertensive medication, previous hyperglycemia, and family history of diabetes [5]. It has been validated to predict the 10-year risk of type 2 diabetes with 78-81% sensitivity and 76-77% specificity [6]. Furthermore, the European Society for the Study of Diabetes and the European Society of Cardiology have recommended the score as a screening tool for type 2 diabetes [6].

Despite its utility, there is often a significant difference between actual risk and self-perceived risk of diabetes among individuals, while little is known about the perceived risk of diabetes in the AMI population. Understanding how AMI patients perceive their diabetes risk and identifying the factors associated with underestimation is essential for the development of effective prevention and treatment strategies in this high-risk group [3, 4].

The primary objective of this study was to assess the discrepancy between diabetes risk perception and the FINDRISC evaluation in post-AMI patients without diagnosed diabetes. This assessment was conducted using data from the epidemiologic Myocardial Infarction Registry Augsburg. The data is population-based and thus well-suited for this analysis due to its comprehensive and unbiased approach. In addition, this study aimed to identify factors associated with the underestimation of drug-treated diabetes risk in the post-AMI population in Germany.

## Methods

### Study population

The study was based on data from a postal follow-up survey (Augsburger Herzinfarkt-Versorgungsstudie 2023) conducted in participants of the Myocardial Infarction Registry Augsburg. This population-based registry was established in 1984 as part of the MONICA project (Monitoring Trends and Determinants in Cardiovascular Disease) as the KORA (Cooperative Health Research in the Region Augsburg) Myocardial Infarction Registry. Since 2021, it runs as Myocardial Infarction Registry Augsburg. It includes patients who have had a clinically confirmed, hospitalized AMI or who had died prehospitally due to ischemic heart disease [7]. The registry covers the city of Augsburg, Germany, and the two adjacent counties of Augsburg and Aichach-Friedberg, with a total population of approximately 680,000.

In April 2023, all survivors of acute or recurrent myocardial infarction admitted to a hospital between 2017 and 2019 (*n* = 1,712) were contacted via a postal questionnaire. A total of 857 patients (50.1%) returned the completed questionnaire. Among the non-responders, 67 patients had passed away, 104 had relocated to an unknown address, and 42 were unwilling or unable to participate. The remaining individuals (*n* = 642) received a reminder by mail but did not respond.

For statistical analysis, the study excluded patients previously diagnosed with diabetes. Additionally, patients lacking complete information on FINDRISC criteria (see 2.2) or self-perceived diabetes risk were omitted from the analysis.

All participants provided written informed consent. The data collection methods received approval from the the ethics committee at the Bavarian Medical Association (Bayerische Landesärztekammer), and the study adhered to the ethical guidelines of the Declaration of Helsinki. The study was registered at the German Register of Clinical Studies (DRKS, project number DRKS00029042).

### Variables of interest

The Augsburger Herzinfarkt-Versorgungsstudie 2023 utilized a questionnaire to assess self-reported information about demographics, health status, history of diabetes, health-related quality of life, symptoms of depression, fatigue, and mental health literacy. This data was linked with existing medical chart data provided by the Myocardial Infarction Registry to enhance the dataset with further socio-economic and clinical data.

Fourty questions were applicable to all participants, while an additional seven targeted those with diagnosed diabetes. For the present analysis, 11 specific questions were selected that included assessments of age, weight, height, waist circumference (female: <80 cm, 80–88 cm,>88 cm; male: <94, 94–102 cm, > 102 cm), physical activity, healthy eating habits, use of antihypertensive medication, previous hyperglycemia, and first or second degree family history of diabetes (first degree: immediate relatives like parents or siblings; second degree: distant relatives like grandparents or aunts/uncles).

Responses to these questions were scored according to the scheme established by Lindström et al. [5], and the FINDRISC score was calculated as the sum of these subscores. The score was used to categorize patients into five risk levels for developing diabetes within the next decade: ≤6 points (very low), 7–11 points (low), 12–14 points (moderate), 15–20 points (high), and > 20 points (very high). According to Lindström et al., the corresponding estimated risk of diabetes in the next 10 years is 1%, 4%, 17%, 33%, and 50%, respectively [5, 8]. Participants were also asked to self-report their perceived risk of developing diabetes if they had not been diagnosed, with response options matching the five FINDRISC categories.

Additional questionnaire items were selected that assessed marital status, education level and mental health. For multivariable analysis, marital status was simplified by combining ‘divorced’ and ‘widowed’ participants with ‘singles’, resulting in two categories: ‘single’ and ‘married’. Education was categorized into several levels (dropped out of school, 9 years, 10 years, 12 years, university degree) and dichotomized into ‘low’ and ‘high’ education categories, using 12 years of schooling as the threshold for higher education for clearer statistical interpretation.

The PHQ-9 (Patient Health Questionnaire-9) score, a screening tool for depressive symptoms based on the DSM-IV (Diagnostic and Statistical Manual of Mental Disorders) system, was also assessed. It included nine items that evaluate the frequency of depressive symptoms experienced over the past two weeks. The total score was categorized into four levels of depression severity (mild, moderate, moderately severe, severe) as defined by Kroenke et al. [9]. For multivariable logistic regression analysis, this score was treated as a continuous variable to provide a nuanced interpretation of mental health’s impact on self-perceived diabetes risk.

Data from participants who completed the questionnaire were integrated with medical chart data to encompass information on smoking history and the clinical course of AMI. The expanded dataset included variables such as previous AMI, type of infarction (ST Elevation Myocardial Infarction, Non-ST Elevation Myocardial Infarction), and therapeutic procedures such as percutaneous transluminal coronary angioplasty (PTCA) and aorto-coronary bypass surgeries. Furthermore, the dataset included the time interval between the occurrence of AMI and the completion of the survey.

### Statistical analysis

The primary outcome of this study was the misperception of drug-treated diabetes risk in AMI patients, defined by the difference between self-assessed diabetes risk and the FINDRISC score. This difference categorized patients into three groups: those who overestimated (positive difference), underestimated (negative difference), or accurately assessed (zero difference) their risk. For statistical analysis, participants who overestimated or accurately assessed their risk were combined into the single group “Overestimation”, based on findings that overestimation is less harmful than underestimation in terms of preventive behaviors [10]. Thus, the comparative analysis focused on the differences between participants who underestimated and those who did not underestimate their risk.

Baseline characteristics were presented as absolute frequencies and percentages for categorical variables and differences in them between the groups were evaluated using chi-squared tests. For continuous variables, differences were assessed using Student’s t-test or the Mann-Whitney U test (non-normally distributed variables), with results presented as means with standard deviations (SD) and medians with interquartile ranges (IQR).

Multivariable logistic regression models were calculated to identify variables significantly associated with the underestimation of drug-treated diabetes risk. Prior to conducting the logistic regression analysis, the model’s assumptions were validated to ensure the reliability and validity of the results (tests for multicollinearity and log-linearity of continuous variables).

The initial logistic regression model included a comprehensive set of variables, including age, sex, BMI, waist circumference, marital status, education level, diet, physical activity, smoking, first and second degree family history of diabetes and second degree family history of diabetes, previous diagnosed elevated blood glucose levels (dichotomous), antihypertensive drug use, PHQ9 score (categorical), previous AMI, type of infarction, PTCA, aorto-coronary bypass and time span from AMI. A backward elimination process was employed manually, whereby the least significant predictor was removed at each step. This process was continued until only statistically significant predictors remained in the parsimonious model. The results are presented as odds ratios (OR) with 95% confidence intervals (CI) and p values.

To further validate the logistic regression model, a supplementary linear regression analysis was conducted and is detailed in Supplementary Material Table S1. This analysis aimed to confirm the difference between perceived and calculated FINDRISC diabetes risk scores (outcome) by assessing how demographic, clinical, and behavioral factors in the linear regression model contribute to any discrepancies in risk perception.

The analysis was restricted to complete case data. A p-value of less than 0.05 was considered indicative of statistical significance. All statistical analyses were performed using R Studio (Version 2023.12.1 + 402) with the following packages: broom, car, dplyr, ggplot2, glm2, haven and readr.

## Results

**Fig. 1:**
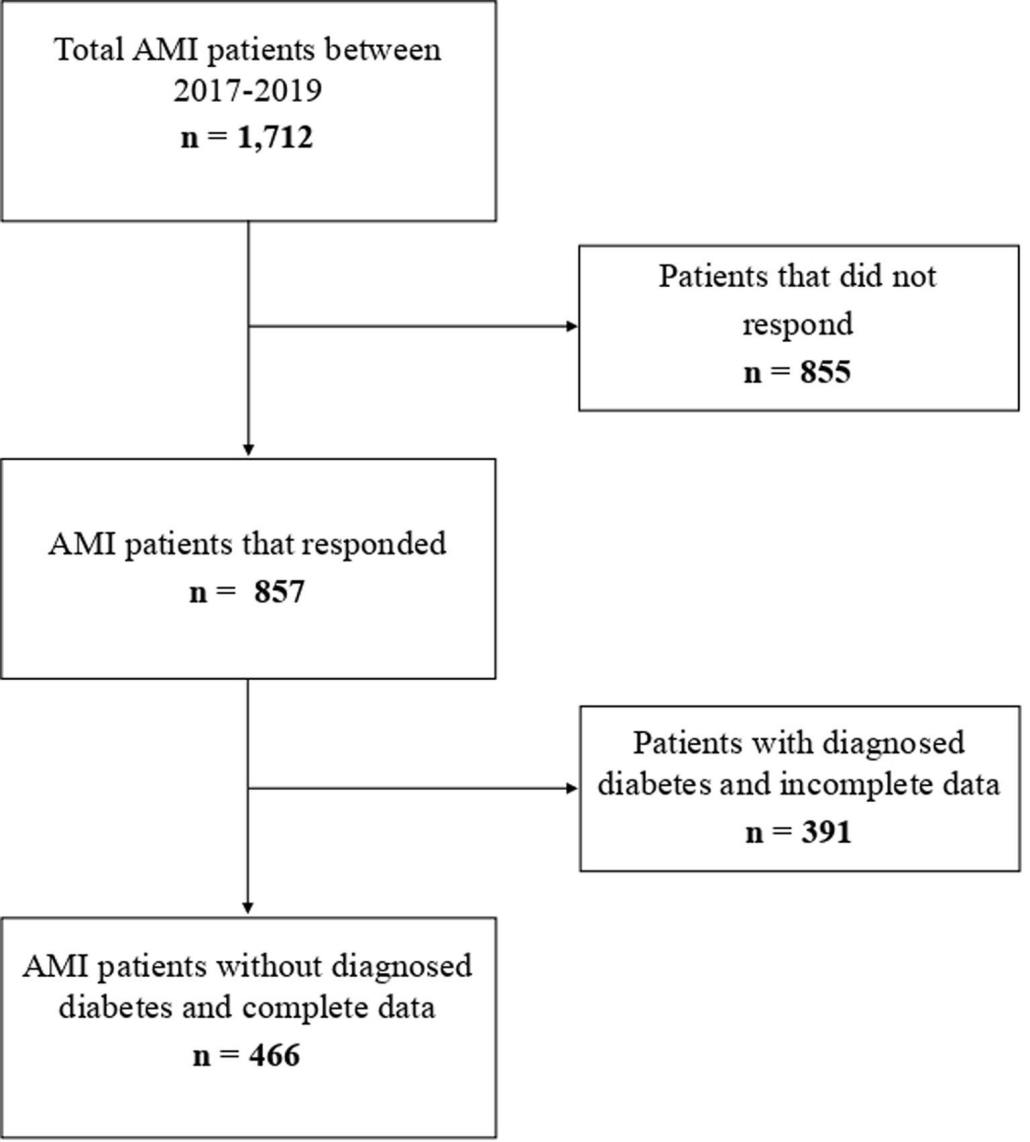
Flowchat showing inclusion and exclusion process after data collection

The survey was completed by 857 AMI patients (Fig. 1). A total of 391 patients were excluded due to being previously diagnosed with diabetes (*n* = 210 [24.5%]) or having missing data on self-perceived diabetes risk and incomplete FINDRISC variables (*n* = 181) (Fig. 1). Among the remaining 466 patients, 110 (23.6%) were female, with an overall mean age of 70.3 years (SD = 11.1 years, Table 1). Notably, 45.1% of participants reported an increased waist circumference (female: 80–88 cm, male: 94–102 cm), and 41% reported a severely increased waist circumference (female: >88 cm, male: >102 cm). The mean BMI was 27.2 (SD = 4.3). Regarding education, 74.5% had less than 12 years of schooling, and 73.2% were married. The majority (87.5%) experienced their first AMI.

**Table 1:** Baseline characteristics information for the non-diabetic sample and stratified for self-perceived vs. FINDRISC risk assessment presented as total number and % or mean and SD or median and IQR

	Total sample *N* = 466	No Under-estimation *N* = 194	Under-estimation *N* = 272	*P* Value	N*
Demographics					
Age (mean, SD)	70. (11.1)	68.5 (11.8)	71.6 (10.5)	0.003	466
Female	110(23.6)	41(21.1)	69(25.4)	0.342	466
BMI (mean, SD)	27.2 (4.3)	26.0 (4.2)	28.1 (4.1)	< 0.001	466
Waist circumference				< 0.001	466
Normal	65(14.0)	47(24.2)	18(6.6)		
Increased	210(45.1)	90(46.4)	120(44.1)		
Severely increased	191(41.0)	57(29.4)	134(49.3)		
Marital status				0.114	466
Married	341(73.2)	134(69.1)	207(76.1)		
Single	125(26.8)	60(30.9)	65(23.9)		
Education/ Lifestyle					
Education				0.008	463
Low Education	345(74.5)	131(67.7)	214(79.3)		
High Education	118(25.5)	62(32.1)	56(20.7)		
Current or former smoker	276(59.5)	113(58.3)	163(60.4)	0.716	464
Daily physical activity ≥ 30 min	413(88.6)	173(89.2)	240(88.2)	0.867	466
Whole foods daily	307(65.9)	127(65.5)	180(66.2)	0.951	466
Clinical Characteristics					
Elevated blood Glucose ever	100(21.5)	21(10.8)	79(29.0)	< 0.001	466
Antihypertensive medication	386(82.8)	149(76.8)	237(87.1)	0.005	466
High blood lipids	261(56.0)	109(56.2)	152(55.9)	1	466
1st degree diabetes family history	118(25.3)	20(10.3)	98(36)	< 0.001	466
2nd degree diabetes family history	28(6.0)	10(5.2)	18(6.6)	0.647	466
Acute event					
Days between AMI (median, IQR) and follow-up survey	1786.5 (1471.2–2034.2)	1798.5 (1467.2–2058.0)	1780.0 (1475.0–2011.2)	0.52	466
Previous AMI	58(12.5)	22(11.3)	36(13.3)	0.629	465
STEMI	197(45)	83(45.1)	114(44.9)	1	438
PTCA	397(85.4)	167(86.1)	230(84.9)	0.654	466
Aorto-Coronary Bypass	43(9.4)	17(8.8)	26(9.7)	0.861	460
Self-Perceived Diabetes Risk				< 0.001	466
Very Low	130(27.9)	12(6.2)	118(43.4)		
Low	225(48.3)	117(60.3)	108(39.7)		
Moderate	98(21)	53(27.3)	45(16.5)		
High	11(2.4)	10(5.2)	1(0.4)		
Very High	2(0.4)	2(1.0)	0(0)		
FINDRISC				< 0.001	466
Very Low	30(6.4)	30(15.5)	0(0)		
Low	194(41.6)	124(63.9)	70(25.7)		
Moderate	101(21.7)	29(14.9)	72(26.5)		
High	124(26.6)	9(4.6)	115(42.3)		
Very High	17(3.6)	2(1.0)	15(5.5)		
Depressive Disorder				0.387	449
Absent/minimal	253 (56.3)	106 (56.4)	147 (56.3)		
Mild/Subthreshold	150 (33.4)	61 (32.4)	89 (34.1)		
Moderate	39 (8.7)	18 (9.6)	21 (8.0)		
Pronounced	5 (1.1)	1 (0.5)	4 (1.5)		
Severe	2 (0.4)	2 (1.1)	0 (0.0)		

*Number of cases with valid information

AMI = acute myocardial infarction; BMI = Body Mass Index; IQR = Interquartile range; MI = Myocardial Infarction; PTCA = Percutaneous transluminal coronary angioplasty; SD = Standard deviation; STEMI = ST Elevation Myocardial Infarction

The mean FINDRISC score was 12.4 (SD 4.2), 6.4% of participants were classified at very low risk, 42% at low risk, 22% at moderate risk, 27% at high risk, and 4% at very high risk for developing diabetes within 10 years (Table 1). Self-perception of diabetes risk revealed that 28% perceived themselves at very low risk, 48% at low, 21% at moderate, 2% at high, and less than 1% at very high risk (Table 1).

Overall, 194 patients did not underestimate their drug-treated diabetes risk, while 272 underestimated their personal risk (Table 1). Patients who underestimated their diabetes risk were older, had higher BMI and waist circumference, were more frequently on antihypertensive medication, had lower education levels, and reported more frequently previously elevated blood glucose levels or had more often a direct or indirect family history of diabetes (Table 1). Table 2 displays the baseline characteristics of patients with diabetes at follow-up and the groups of patients without diabetes, who were excluded due to missing values on one or more of the variables needed to calculate the FINDRISC score. It shows that, apart from education, there were no significant differences between the group of patients excluded due to missing values and the group of patients that was used for the analysis.

**Table 2:** Baseline characteristics for the patients included into the analysis, for the diabetic sample and for the non-diabetic patients excluded due to missing values. Data is presented as total number and % or mean and SD or median and IQR. Information for the non-diabetic sample and stratified for self-perceived vs. FINDRISC risk assessment presented as total number and % or mean and SD or median and IQR

	Total sample *N* = 466	Patients excluded due to missing values *N* = 181	Patients with Diabetes *N* = 210	*P* Value	N*	*P* Value– Total sample vs. patients excluded due to missing values	*P* Value– Total sample vs. patients with diabetes
Demographics							
Age (mean, SD)	70.3 (11.1)	72.1 (10.8)	70.9 (10.3)	0.15	857	0.053	0.467
Female	110 (23.6)	39 (21.5)	43 (20.5)	0.634	857	0.650	0.424
BMI (mean, SD)	27.2 (4.3)	27.0 (3.7)	29.5 (5.2)	< 0.001	839	0.467	< 0.001
Waist circumference				< 0.001	839	0.337	< 0.001
Normal	65 (13.9)	30 (18.5)	17 (8.5)				
Increased	210 (45.1)	66 (40.7)	65 (32.5)				
Severely increased	191 (41.0)	66 (40.7)	118 (59.0)				
Marital status				0.397	853	0.684	0.206
Married	341 (73.2)	126 (71.2)	143 (68.1)				
Single	125 (26.8)	51 (28.8)	67 (31.9)				
Education/Lifestyle							
Education				0.070	846	0.029	0.812
Low Education	345 (74.5)	147 (83.1)	156 (75.7)				
High Education	118 (25.5)	30 (16.9)	50 (24.3)				
Current or former smoker	276 (59.5)	106 (58.9)	136 (65.1)	0.331	853	0.961	0.197
Daily physical activity ≥ 30 min	413 (88.6)	162 (92.6)	162 (77.9)	< 0.001	849	0.187	< 0.001
Whole foods daily	307 (65.9)	117 (65.7)	140 (67.3)	0.926	852	1	0.784
Clinical Characteristics							
Elevated blood Glucose ever	100 (21.5)	26 (14.7)	198 (95.7)	< 0.001	850	0.069	< 0.001
Antihypertensive medication	386 (82.8)	147 (82.6)	189 (90.4)	0.028	853	1	0.014
High blood lipids	261 (56.0)	93 (51.4)	141 (67.1)	0.004	857	0.330	0.008
1st degree diabetes family history	118 (25.3)	29 (16.0)	100 (47.6)	< 0.001	857	0.015	< 0.001
2nd degree diabetes family history	28 (6.0)	11 (6.1)	32 (15.2)	< 0.001	857	1	< 0.001
Acute event							
Days between AMI (median, IQR) and follow-up survey	1786.5 (1471.2–2034.2)	1713.0 (1463.0–2066.0)	1799.0 (1523.5–2022.8)	0.435	857	0.608	0.346
Previous AMI	58 (12.5)	20 (11.0)	36 (17.1)	0.153	856	0.716	0.133
STEMI	197 (45.0)	64 (38.3)	67 (34.0)	0.025	802	0.166	0.012
PTCA	397 (85.2)	151 (83.4)	183 (87.1)	0.712	857	0.702	0.662
Aorto-Coronary Bypass	43 (9.3)	20 (11.1)	18 (8.7)	0.697	848	0.599	0.886
Self-Perceived Diabetes Risk					499	0.915	-
Very Low	130 (27.9)	9 (27.3)	-				
Low	225 (48.3)	18 (54.5)	-				
Moderate	98 (21.0)	5 (15.2)	-				
High	11 (2.4)	1 (3.0)	-				
Very High	2 (0.4)	0 (0.0)	-				
FINDRISC				< 0.001	813	0.084	< 0.001
Very Low	30 (6.4)	15 (10.1)	1 (0.5)				
Low	194 (41.6)	70 (47.0)	9 (4.5)				
Moderate	101 (21.7)	35 (23.5)	15 (7.6)				
High	124 (26.6)	27 (18.1)	109 (55.1)				
Very High	17 (3.6)	2 (1.3)	64 (32.3)				
Depressive Disorder				0.003	812	0.463	< 0.001
Absent/minimal	253 (56.3)	89 (53.6)	91 (46.2)				
Mild/Subthreshold	150 (33.4)	53 (31.9)	65 (33.0)				
Moderate	39 (8.7)	18 (10.8)	24 (12.2)				
Pronounced	5 (1.1)	5 (3.0)	12 (6.1)				
Severe	2 (0.4)	1 (0.6)	5 (2.5)				

*Number of cases with valid information

AMI = acute myocardial infarction; BMI = Body Mass Index; IQR = Interquartile range; MI = Myocardial Infarction; PTCA = Percutaneous transluminal coronary angioplasty; SD = Standard deviation; STEMI = ST Elevation Myocardial Infarction

In the group of the 466 patients included into the analysis, a comparison between self-perceived and FINDRISC evaluation indicated that one-third of participants (33%) correctly estimated, 9% overestimated, and 58% underestimated their risk (Table 3). Of 141 patients at high or very high risk according to the FINDRISC score, 92% (*n* = 130) showed an underestimation of their risk (Table 3).

**Table 3:** Comparison self-perceived diabetes risk vs. FINDRISC score Color coding indicates correct self-perception (bold, *n* = 154), overestimation (italic, *n* = 40), and underestimation (bolditalic, *n* = 272) of diabetes risk

Perceived Diabetes Risk	FINDRISC					
	Very low	Low	Moderate	High	Very high	Total
MALE + FEMALE						
Very low	**12**	***70***	***28***	***17***	***3***	130
Low	*15*	**102**	***44***	***60***	***4***	225
Moderate	*3*	*21*	**29**	***38***	***7***	98
High	*0*	*1*	*0*	**9**	***1***	11
Very High	*0*	*0*	*0*	*0*	**2**	2
Total	30	194	101	124	17	466
MALE Perceived Diabetes Risk						
Very low	**11**	***49***	***21***	***13***	***3***	97
Low	*10*	**82**	***33***	***42***	***3***	170
Moderate	**2**	*16*	**24**	***33***	***5***	80
High	*0*	*0*	*0*	**6**	***1***	7
Very High	*0*	*0*	*0*	*0*	**2**	2
Total	23	147	78	94	14	356
FEMALE						
Very low	**1**	***21***	***7***	***4***	***0***	33
Low	*5*	**20**	***11***	***18***	***1***	55
Moderate	*1*	*5*	**5**	***5***	***2***	18
High	*0*	*1*	*0*	**3**	***0***	4
Very High	*0*	*0*	*0*	*0*	**0**	0
Total	7	47	23	30	3	110

The multivariable logistic regression model (*n* = 410) confirmed the results of the descriptive statistics, see Table 4. It revealed that older age (OR = 1.06 [1.03–1.08]; *p* < 0.001), BMI (OR = 1.13 [1.04–1.23]; *p* = 0.0042), higher waist circumference (high: OR = 2.83 [1.38–6.00], *p* = 0.0055; very high: OR = 2.93 [1.19–7.43], *p* = 0.0212), positive familial diabetes history (first degree: OR = 6.49 [3.62–12.23], *p* < 0.001; second degree: OR = 2.70 [1.11–6.97], *p* = 0.0326), use of antihypertensive medication (OR = 2.03 [1.13–3.68]; *p* = 0.0181) and history of hyperglycemia (OR = 3.88; [2.17–7.22]; *p* < 0.001) were associated with underestimation of drug-treated diabetes risk. In contrast, having a higher education level (OR = 0.55 [ 0.33–0.93]; *p* = 0.0268) indicated an inverse association with the risk of underestimation. The linear regression model confirmed these significant predictors (Supplemental Table S1).

**Table 4:** Results of the multivariable logistic regression model results of the parsimonious model after backward selection examining the association between factors associated with underestimation of diabetes risk according to FINDRISC score

	OR (95%)	*P* value
Characteristics		
Age	1.06 [1.03–1.08]	< 0.001
Male	0.86 [0.48–1.50]	0.5866
BMI	1.13 [1.04–1.23]	0.0042
Higher Education	0.55 [0.33–0.93]	0.0268
Increased Waist circumference	2.83 [1.38–6.02]	0.0055
Severely increased Waist circumference	2.91 [1.17–7.36]	0.0212
1st degree Family History	6.49 [3.62–12.23]	< 0.001
2nd degree Family History	2.70 [1.11–6.97]	0.0326
Use of antihypertensives	2.03 [1.13–3.68]	0.0181
Elevated Blood Glucose (ever)	3.88 [2.17–7.22]	< 0.001

The figures in bold indicate significant determinants.

OR = Odds Ratio; BMI = Body Mass Index

## Discussion

This study analyzed responses from 466 AMI patients from the Augsburg Myocardial Infarction Registry, showing significant differences between self-perceived and calculated drug-treated diabetes risk based on the FINDRISC score. Notably, 58% of participants underestimated their drug-treated diabetes risk, especially those with older age, higher BMI, greater waist circumference, previously elevated glucose levels and a family history of diabetes. These patients reported lower educational level and to be on antihypertensive medication. In contrast, higher education appeared to provide a protective association against underestimation.

Consistent with the findings of this study focusing on AMI patients, previous research in the general population have reported relatively low awareness of diabetes risk, highlighting a disconnect between individual perceptions and actual risk factors [11–14].

In contrast, previous KORA studies that focused on a healthy population without diagnosed diabetes and AMI found that individuals typically accurately estimate or overestimate their diabetes risk when they are aware of risk factors such as increased waist circumference, parental diabetes, and higher weight [11, 14]. However, these factors were associated with underestimation in the AMI-specific sample, demonstrating a significant deviation in risk perception between general population and CVD-affected patients.

Moreover, this study’s result aligns with findings from Kilkenny et al. [15], who found a counterintuitive aspect of disseminating health knowledge: individuals with or at risk for CVD often have less knowledge about diabetes and CVD risk factors than their low-risk counterparts. This paradox highlights a critical gap in health education, which is particularly alarming given that these high-risk individuals are the ones who most need to understand and effectively manage their risk factors.

Despite these differences, a consistent trend across both the general population and AMI patients is the positive association between higher educational levels and more accurate diabetes risk perception [11, 14]. This suggests that understanding and awareness of diabetes risk factors could be significantly improved through educational interventions, e.g. by physicians [16]. In addition, sex was not found to be a significant factor in under- or overestimating diabetes risk in both the general population [11, 12, 14] and the AMI patients. This observation may be due to the higher prevalence of males in the study’s sample that may have obscured any sex-specific differences in risk perception. However, the proportion of women with myocardial infarction is lower than for men, which is reflected in the study sample.

A systematic review on the perception of inherited risk in type 2 diabetes found that those who were aware of genetic risk as a causal explanation were more concerned about developing type 2 diabetes, while those who were unaware of the genetic link were less concerned about developing the disease [17]. This finding underlines the need for more education about potential risk factors for diabetes especially in those with a family history of diabetes. Furthermore, most studies agree that older age is associated with diabetes risk underestimation [11–14]. This may be due to the lower lifetime risk of diabetes observed in older individuals [20]. Another potential explanation is that older individuals may have less knowledge about health and diabetes risk factors compared to younger individuals.

The presence of significant predictors for diabetes across populations, such as age, BMI, glucose levels and hypertension, highlights the universal importance of these factors in drug-treated diabetes risk perception [18]. The Robert Koch Institute’s nationwide telephone survey also emphasized the strong need for information on lifestyle changes, health promotion, and disease prevention among people without diabetes, suggesting a gap in public health messaging [19].

In addition, physician communication about diabetes risk has been shown to significantly influence perceptions, increasing the likelihood that individuals view themselves at an increased risk [11]. This finding suggests an important intervention target in the AMI population, where physician communication could significantly improve patients’ risk perceptions.

In the present study, the FINDRISC score was selected to assess diabetes risk prediction. This score has been noted for its accuracy in predicting the rate of incident diabetes across age subgroups without significant differences [20]. It is important to note, however, that the FINDRISC score has shown variability in its predictions, particularly overestimating risk in individuals with higher waist circumferences and underestimating risk in individuals with healthier waist measurements [20]. In addition, the FINDRISC prediction has been associated with cardiovascular events [21, 22], an association that is plausible given the well-established association between diabetes and CVD [23]. This aspect of FINDRISC score’s performance may partially explain the tendency of the AMI patient sample to underestimate their drug-treated diabetes risk, especially since the model relies on a limited set of self-reported responses that may not fully capture the confounding effects of post-AMI metabolic changes.

The QDScore (QDiabetes Score) is a similar diabetes risk score, recommended by the National Institute for Health and Care Excellence, which includes CVD as a predictive factor [24]. The QDScore indicates that the incorporation of more comprehensive health record data could potentially enhance predictive accuracy, particularly in patients with comorbidities. This leads to the question of whether the inclusion of cardiovascular health factors in diabetes risk models could better serve populations such as the AMI sample, where CVD is prevalent.

### Strengths and limitations

This study’s strengths include the use of a comprehensive dataset from the population-based Myocardial Infarction Registry Augsburg and the well-validated FINDRISC score for drug-treated diabetes risk assessment. The available data on each patient allowed the identification of risk factors in a specific population, providing insights into how AMI patients perceive their diabetes risk compared to the general population.

However, the study is not without limitations. The relatively low response rate of approximately 50% and the non-diabetic patients with missing values on covariables relevant for the FINDRISC score may have introduced a selection bias, potentially affecting the generalizability of the findings. The overlap of risk factors for CVD and diabetes may confound the FINDRISC score’s ability to distinctly estimate drug-treated diabetes risk. Additionally, the study’s concentrated geographic focus in the Augsburg area may limit the generalizability of the findings to other regions or populations with varying socioeconomic backgrounds, cultural factors, and healthcare access. Moreover, the absence of further socioeconomic variables, biomarkers, and detailed cultural factors in the dataset restricts the ability to fully understand the interplay of these factors with diabetes risk perception. Another limitation is that the study relies on self-reported data on lifestyle and measurements, which may introduce reporting biases and inaccuracies, affecting the reliability of the findings.

## Conclusion

This study contributes to the understanding of how AMI patients perceive their risk of developing diabetes. the results reveal a significant discrepancy between the perceived and actual risk levels as defined according to FINDRISC. These findings underscore the need for improved educational strategies to enhance patient understanding of their health status. Specifically, addressing the underlying factors that lead to risk underestimation—such as insufficient educational resources, suboptimal communication from healthcare providers, and an inadequate understanding of risk factors that play a role in developing diabetes, including age, BMI, waist circumference, blood glucose levels, hypertension, and genetics—is crucial. Enhancing diabetes risk awareness among AMI patients is important, not only for improving their own health outcomes but also for reducing the burden on healthcare systems. Educational preventive measures in daily practice facilitate the alignment of patients’ perceptions with their actual risk.

Future initiatives should focus on the development of comprehensive educational tools and the implementation of targeted communication strategies that are accessible and effective across diverse patient populations, with a particular focus on those with lower educational backgrounds. Additionally, expanding research on AMI patients to include a wider range of sociodemographic and cultural variables can enhance the generalizability of these findings and help develop interventions to meet the specific needs of different communities.

## Electronic supplementary material

Below is the link to the electronic supplementary material.

Supplementary Material 1

## Data Availability

The datasets generated during and/or analysed during the current study are not publicly available due to data protection aspects but are available in an anonymized form from the corresponding author on reasonable request.

## Abbreviations

AMI: Acute Myocardial Infarction
BMI: Body Mass Index
CI: Confidence Interval
CVD: Cardiovascular Disease
DSM-IV: Diagnostic and Statistical Manual of Mental Disorders (4th Edition)
FINDRISC: Finnish Diabetes Risk Score
IQR: Interquartile Range
PHQ-9: Physical Health Questionnaire- 9
PTCA: Percutaneous transluminal coronary angioplasty
SD: Standard Deviation
QDScore: QDiabetes Score
