# Supplementary material for: Diabetes mellitus risk in post-myocardial infarction patients: FINDRISC versus self-assessment—a cross sectional study
Source: Cardiovasc Diabetol. 2025 Jan 18;24:23. doi: 10.1186/s12933-024-02551-1 (PMC11743004; doi:10.1186/s12933-024-02551-1)
Supplement: Supplementary file 1 — Supplementary Material 1 [file 12933_2024_2551_MOESM1_ESM.docx]

**Table S1** Linear Regression Analysis of Risk Difference Between Self-Perveived Diabetes Risk and FINDRISC Score

*The results of the linear regression model assessing the determinants impacting the difference between self-perceived diabetes risk and calculated FINDRISC categories are presented as coefficients and p values. The FINDRISC risk and perceived risk categories are numerically coded as 1 (very low), 2 (low), 3 (moderate), 4 (high), and 5 (very high). Coefficients represent the direction and magnitude of each determinant's impact. A positive coefficient indicates a tendency to overestimate risk relative to the FINDRISC assessment, whereas a negative coefficient indicates underestimation.*

|  | Coefficient | P value |  |  |
| --- | --- | --- | --- | --- |
| Characteristics | |  |  |  |
| *Age* | | **-0.02891** | **<0.001** |  |
| *Male* | | 0.06212 | 0.545 |  |
| *BMI* | | **-0.04459** | **0.001** |  |
| *Married* | | - 0.1611 | 0.08 |  |
| *Higher Education* | | **0.306** | **0.002** |  |
| *Waist circumference high* | | **-0.563** | **<0.001** |  |
| *Waist circumference very high* | | **-0.6396** | **<0.001** |  |
| *Healthy Diet* | | **0.2441** | **0.005** |  |
| *Physical Activity* | | 0.1795 | 0.154 |  |
| *Smoker (ever)* | | -0.0702 | 0.411 |  |
| *1^st^ degree Diabetes Family History* | | **-0.9488** | **<0.001** |  |
| *2^nd^ degree Diabetes Family History* | | **-0.7311** | **<0.001** |  |
| *Days from MI* | | -0.000008 | 0.948 |  |
| *Antihypertensives* | | **-0.3496** | **0.001** |  |
| *Elevated Blood Glucose (ever)* | | **-0.6703** | **<0.001** |  |
| *High Blood Lipids* | | **0.1916** | **0.019** |  |
| *Previous AMI* | | -0.1202 | 0.337 |  |
| *Type of infarction - STEMI* | | -0.0473 | 0.564 |  |
| *PTCA* | | 0.0531 | 0.287 |  |
| *Aorto-Coronary Bypass* | | 0.1684 | 0.318 |  |
| *PHQ-9* | | 0.01881 | 0.416 |  |
| The figures in bold indicate significant determinants. | | | |  |
